# Effectiveness of Serious Gaming During the Multidisciplinary Rehabilitation of Patients With Complex Chronic Pain or Fatigue: Natural Quasi-Experiment

**DOI:** 10.2196/jmir.9739

**Published:** 2018-08-15

**Authors:** Miel AP Vugts, Margot CW Joosen, Agali Mert, Aglaia ME Zedlitz, Hubertus JM Vrijhoef

**Affiliations:** ^1^ Tranzo Scientific Center for Care and Welfare Tilburg School of Social and Behavioral Sciences Tilburg University Tilburg Netherlands; ^2^ Mert Medical Utrecht Netherlands; ^3^ Leiden Institute for Brain and Cognition Department of Health Medical and Neuropsychology Leiden University Leiden Netherlands; ^4^ Department of Family Medicine and Chronic Care Vrije Universiteit Brussel Brussels Belgium; ^5^ Department of Patient & Care Maastricht University Medical Center Maastricht Netherlands; ^6^ Panaxea BV Amsterdam Netherlands

**Keywords:** behavioral intervention, chronic pain, serious games, musculoskeletal disease, rehabilitation, therapy

## Abstract

**Background:**

Current evidence for the effectiveness of specialist multidisciplinary programs for burdensome chronic pain and functional somatic syndromes drives the effort to improve approaches, strategies, and delivery modes. It remains unknown to what extent and in what respect serious gaming during the regular outpatient rehabilitation can contribute to health outcomes.

**Objective:**

The objectives of our study were to determine the effect of additional serious gaming on (1) physical and emotional functioning in general; (2) particular outcome domains; and (3) patient global impressions of change, general health, and functioning and to determine (4) the dependency of serious gaming effects on adherence.

**Methods:**

We conducted a naturalistic quasi-experiment using embedded qualitative methods. The intervention group patients received an additional guided (mindfulness-based) serious gaming intervention during weeks 9-12 of a 16-week rehabilitation program at 2 sites of a Dutch rehabilitation clinic. Simultaneously, 119 control group patients followed the same program without serious gaming at 2 similar sites of the same clinic. Data consisted of 10 semistructured patient interviews and routinely collected patient self-reported outcomes. First, multivariate linear mixed modeling was used to simultaneously estimate a group effect on the outcome change between weeks 8 and 16 in 4 primary outcomes: current pain intensity, fatigue, pain catastrophizing, and psychological distress. Second, similar univariate linear mixed models were used to estimate effects on particular (unstandardized) outcomes. Third, secondary outcomes (ie, global impression of change, general health, functioning, and treatment satisfaction) were compared between the groups using independent t tests. Finally, subgroups were established according to the levels of adherence using log data. Influences of observed confounding factors were considered throughout analyses.

**Results:**

Of 329 eligible patients, 156 intervention group and 119 control group patients (N=275) with mostly chronic back pain and concomitant psychosocial problems participated in this study. Of all, 119 patients played ≥75% of the game. First, the standardized means across the 4 primary outcomes showed a significantly more favorable degree of change during the second part of the treatment for the intervention group than for the control group (beta=−0.119, SE=0.046, *P*=.009). Second, the intervention group showed a greater outcome change in depressive mood (b=−2.748, SE=1.072, *P*=.011) but not in “insufficiency” or concentration problems. Third, no significant group effects on secondary outcomes were found. Fourth, adherence was generally high and invariant.

**Conclusions:**

The findings of this study suggest a very small favorable average effect on relevant health outcomes of additional serious gaming during multidisciplinary rehabilitation. The indication that serious gaming could be a relatively time-efficient component warrants further research into if, when, how, and for which patients serious gaming could be cost-effective in treatment and why.

**Trial Registration:**

Netherlands Trial Registry NTR6020; http://www.trialregister.nl/trialreg/admin/rctview.asp?TC=6020 (Archived by WebCite at http://www.webcitation.org/71IIoTXkj)

## Introduction

### Background

In a European survey, it has been estimated that 77% of patients with chronic pain (CP) do not access specialist treatments and 40% cannot effectively control their pain [1]. CP is defined by the presence of pain beyond a usual 3- to 6-month duration of organic recovery that may, but does not have to, have an organic cause [2]. Functional somatic syndromes (FSS) are characterized by a persistent pattern of bodily symptoms (ie, pain, fatigue, tinnitus, bowel complaints, and palpitations) for which adequate examination does not reveal sufficiently explanatory specified pathology [3]. Both classifications include, among others, fibromyalgia, chronic low back pain, and irritable bowel syndromes. Global prevalence estimates vary with location and case criteria (severity and disability) and are generally considered high (7%-64% for CP, 3%-43% for tinnitus, 14%-33% for noncardiac chest pain, and 1% for chronic fatigue syndrome) [4-10]. In the absence of satisfactory biomedical solutions, biopsychosocial interventions are offered for improving the physical and emotional functioning [2,3]. A major challenge is to identify which behavior change intervention approaches [11], techniques [12], and delivery modes (eg, computer based) are most accessible and (cost-)effective for certain patients in certain health care settings [13,14]. Herein, a contribution may be made by the pragmatic effectiveness evaluation of a serious gaming intervention (ie, LAKA) during multidisciplinary rehabilitation for patients with emotional or role dysfunctions in association with CP or fatigue [15]. It is hitherto unknown whether, to what extent, and in what respect serious games complement other treatment modes in facilitating intervention effects that are meaningful for patients when offered in addition to other modes of treatment as in regular outpatient multidisciplinary rehabilitation.

### Existing Treatment Gaps

National and international guidelines consider various behavioral interventions to be evidence based, but they change with developing insights into the various CP or FSS conditions [16,17]. Intensive multidisciplinary rehabilitation programs are indicated if locally accessible and (unimodal) conservative medication, minimal self-guided intervention, and physical and psychological therapy do not suffice [3,10,17,18]. Ideally, treatment plans are tailored to individual symptom patterns through interdisciplinary procedures [10]. Supporting evidence from randomized controlled trials (RCTs) consists of, at most, medium-sized effects for biopsychosocial interventions compared with alternative treatments [10,18]. An improvement is sought in the modest additional effects of the multi- or interdisciplinary rehabilitation over other kinds of “unimodal” treatments [10,18-20]. There’s growing evidence for the efficacy of acceptance- and mindfulness-based interventions, which can be included in the multi- or interdisciplinary rehabilitation [10,11]. Rather than addressing certain presumed maladaptive illness beliefs, this sort of approach aims to cultivate self-awareness, self-regulation, and self-transcendence in response to aversive conditions such as CP or FSS [11,21]. Moreover, behavioral interventions have approximately equivalent effects when delivered via computers or internet, but adherence to such interventions can be disappointing [14,22]. Promotion of motivation and adherence (by professionals) may lead to better therapy outcomes [23] and is likely to be of help to patients when using computer-based programs [22]. Few trials have reported mixed results for the efficacy of varied computer-delivered interventions (ie, mobile phone, automated telephone responding, and online support group) offered in addition to face-to-face intervention to patients with CP [24-26]. Generally, effect studies of biopsychosocial interventions may need to improve in their methodological quality (ie, statistical power, risk of selection and reporting bias) and uniformity (ie, definitions of case and recovery, diagnostic methods, subjective and objective outcome criteria, and program description) [27,28].

### Why Serious Games May Offer a Potential Solution

Serious games, which primarily aim at health benefits, may take the form of a video game [29]. Indeed, how harmful or conducive video gaming is for behavior and health depends on the content (eg, whether it reinforces aggressive or prosocial actions) and context (ie, players and instructional support) of the game [30,31]. Serious games may combine small behavioral and clinical benefits with independent accessibility and standardized content of computer-based interventions as well as unique qualities for learning such as intrinsic motivation, enjoyment, positive affect, sense of presence, and meta-cognition [29,32-34]. Games are a ubiquitous but undefinable cultural phenomenon described as bounded “spaces” physically, imaginarily, or in time apart from ongoing reality, wherein individuals involve voluntarily, create meanings, and develop adaptive capacities, such as sports and rituals [35,36]. Intrinsic motivation, as in games, is beneficial for learning quality [37]. It has been hypothesized that behavioral change is strengthened by engagement qualities triggered by storytelling, fantasy, and interactivity in serious games [33]. In the fields of mental health care and rehabilitation, gaming, motion capturing, and virtual reality technologies potentially support the treatment of various well-known conditions such as depression, anxiety, phobias, poststroke, and acute pain [38-42]. After serious gaming, debriefing may be offered to facilitate the transfer of patient experiences into targeted individual learning results [43]. Thus, previous studies have shared the idea of (subtle) positive moderation of treatment effects because of distinctive beneficial motivational qualities triggered by features of serious games. However, adequately powered studies on the comparative effects of games for health are lacking in general, and little to nothing is known about their complementary effectiveness in regular health care contexts, such as multidisciplinary rehabilitation [44]. Moreover, patient adherence to serious gaming when deployed in practice and its influence on outcomes require empirical assessment in effectiveness evaluation [45].

### A Mindfulness Approach to Serious Gaming in Multidisciplinary Rehabilitation

Serious gaming can be a complementary modality that strengthens mindfulness-based modules in treatments like multidisciplinary rehabilitation. Adopting a mindfulness approach to serious gaming deviates from an approach wherein particular antecedent cognitions of health behaviors are targeted [33]. Mindfulness approaches offer mental training principles (ie, focused attention, open monitoring, or ethical enhancement) for promoting (1) a temporary state of nonjudgmental, nonreactive, present-centered attention and awareness (self-awareness); (2) a capacity to effectively modulate behavior (self-regulation); and (3) a positive relationship between self and others, transcending self-focused needs and increasing prosocial characteristics (self-transcendence) [21]. A complementary role of mindfulness-based serious gaming might not necessarily be to facilitate mental training in patients over prolonged periods, but rather to promote independent practicing by any (other) means in the context of daily life. Plausibly, mental training objectives can be temporarily achieved in conjunction with gaming [46], but it can contradict an obsessive drive that could characterize long-term and frequent video gaming [47]. Over longer durations, individuals may apply mental training principles independently in various ways, depending on behavioral factors (recollection of instructions, intent, habit) [21]. Change techniques (eg, commitment to change, action planning, drawing attention to discrepancies between behavior and goals, noncontingent praise, performance instructions, self-monitoring, and salient feedback on behavior) as well as emotional and social consequences, reduction of negative emotions, values affirmation, etc [12], for (novice) mental training activities could be integrated (via an “Avatar” role) into a serious game. From this line of argument, it was proposed that a short serious gaming intervention adds to the effectiveness of a mindfulness-based approach during multidisciplinary rehabilitation for (subtly) better effects on relevant health outcomes in patients with CP or FSS.

### Objectives

In this study, we investigated the effectiveness of serious gaming as a complement to the multidisciplinary rehabilitation of patients with CP or FSS. The selection of health outcomes was guided by a field consensus on the relevance of physical and emotional functioning, patients’ global impression of improvement, and negative effects [48]. The primary objective was to determine the effect of additional serious gaming on multiple domains of physical and emotional functioning simultaneously. Secondary objectives were to understand which outcome domains are particularly affected, positively or negatively, by serious gaming during rehabilitation and whether serious gaming affects patient’s global impressions of change, general health, and functioning. The final objective was to determine whether outcomes of serious gaming are dependent on adherence. Adherence is defined as the extent to which patients expose themselves, in terms of content, frequency, and duration, to the “hard core” of a serious gaming intervention—playing a serious game and attend the debriefing. The following were the research questions:

1. To what extent does an additional serious gaming intervention affect a change in patients’ physical and emotional functioning during regular multidisciplinary rehabilitation?
2. Regarding which particular domain(s) of physical and emotional functioning does an additional serious gaming intervention affect outcome change during multidisciplinary rehabilitation?
3. To what extent does an additional serious gaming intervention during multidisciplinary rehabilitation affect patients’ impressions of change, subjective health and functioning, and satisfaction with treatment?
4. To what extent is the degree of effectiveness dependent on levels of adherence?

## Methods

### Study Design

A protocol for this embedded experimental mixed-methods study was registered (preresults) in the Dutch trial register (NTR6020), previously published in detail [15], and followed accordingly. General information and important executive details relevant to the present objectives are discussed here. In the absence of a legal obligation for medical ethics review, the protocol was reviewed for the protection of patients’ rights in accordance with the letter and reasoning of applicable legislation and research practice and endorsed by the Psychological Ethics Committee of the Tilburg School of Social Sciences (EC-2016.25t). The study design sorts with the nature of multidisciplinary rehabilitation, which is complicated by tailoring, multiple interacting components, and outcome multidimensionality [45]. Quantitative methods were prioritized for assessment purposes. The two-armed naturalistic quasi-experiment was set up pragmatically, comprising an intervention group of patients who received an additional serious gaming intervention offered during weeks 9-12 of a standardized 16-week rehabilitation program at 2 sites of a Dutch rehabilitation clinic. Simultaneously, an approximately equal number of control group patients followed the same program without serious gaming, as usual, at 2 similar sites of the same clinic (from February 2016 to January 2017). Concurrently collected qualitative data were first used to refine hypotheses blind to trial outcomes and later for triangulation and post hoc explanation of quantitative results.

### Setting, Recruitment, and Data Sources

The convenient selection of control sites aimed for homogeneity across the study groups. The 4 participating sites were located in the south of the Netherlands, where multidisciplinary biopsychosocial rehabilitation, but not serious gaming, is covered under basic health care insurance. In view of ecological validity, all patients with a regular physician indication for multidisciplinary rehabilitation who completed the first 8 weeks of rehabilitation were considered eligible for this study. From the beginning of the second half of their rehabilitation program (July-November 2016), patients were consented by their direct care providers. This timing was chosen for patient convenience and optimal response. To lower the risk of selection bias, patient recruitment was closely monitored through regular site visits. Consent was requested for the processing of patients’ codified clinical diagnostic and outcome data and, perhaps, being contacted for an interview. Outcome data consisted of patients’ routine outcome monitoring administered by the clinic through a standardized Web-surveying procedure at the baseline (t0), intermediate (t1: after 8 weeks of treatment), and posttreatment (t2: after 16 weeks of treatment). Only intervention group candidates were requested to answer feedback questions through the same familiar Web-survey procedure immediately after their debriefing session.

To avoid bias by inflicting outcome expectations in patients as subjects and outcomes assessors, information letters did not contain statements about presumed effects of serious gaming or parallel group comparison. After serious gaming, feedback data were made available to the researchers to support the concurrent qualitative research, but routinely collected clinical (diagnostic and outcome) data were not. In this way, data management served to prevent the risk of biased interpretation through breaching the protocoled sequence of hypotheses refinement and quantitative testing.

### Patients

Based on physical and psychological examination results and clinical interviews, physicians indicated eligibility for multidisciplinary rehabilitation treatment based on the following inclusion criteria. The eligible patients were between 18-67 years of age, had pain for more than 6 months or fatigue complaints or musculoskeletal disease for more than 3 months, had no indication for another more cost-effective treatment, and had concomitant psychosocial problems. The exclusion criteria were as follows: patients with psychiatric symptoms that are not adequately controlled, a marked risk of psychological decompensation through a rehabilitation treatment, language or communication problems that make it impossible to follow rehabilitation, or demonstrable inability to change behavior (eg, due to personality disorders, third party liabilities, or otherwise). Notably, no additional computer literacy criteria were applied for participation in this study.

### Interventions

Both study groups received an intensive 16-week biopsychosocial multidisciplinary rehabilitation program with a particular focus on well-being and social role participation [49] (Table 1). Under the supervision of a rehabilitation physician, patients received on average 100 hours of treatment in either one-on-one or in group settings from a team of 2 physiotherapists and 2 registered psychologists with a master’s degree. Weekly intensity varied between 3 and 7 hours, decreasing with an increase in social role participation throughout the program. Overall, patients received 38% physiotherapy, 30% mindfulness approaches, 23% (other kinds of) psychotherapy, and 9% of activating and counseling in social role participation.

**Table 1:** Overview of program components offered during the first and second halves of the regular multidisciplinary rehabilitation program.

Components offered	Weeks 1-8	Weeks 9-16
Physical therapy	Graded activity (group) Exercise therapy Physiotherapy^a^ Education (lifestyle, pain physiology)	Graded activity (group) Exercise therapy
Psychotherapy approaches	Coping with stress Extinction of fear-avoidance beliefs^a^ Cognitive restructuring^a^ Eye movement desensitization^a^ Mentalization techniques^a^	Coping with stress Cognitive restructuring
Activating and counseling in social role participation	Work and health (education and counseling) Social skills	
Mindfulness interventions	Rationale Psychological well-being assessment Mental training Additional 2-day course (mental training skills)	Mental training

^a^Allocation depended on examination results.

The various interventions were centrally assigned, based on individual examination results for physical status, psychological and posttraumatic distress, coping, cognition, and well-being. In this study, the strategies used to promote health behavior were as follows: shaping knowledge about antecedents and health consequences, goal setting and feedback, social support, exposure, behavioral repetition and substitution, skills training (in relaxation, social skills, and mental training), and identity development (ie, cognitive restructuring and values affirmation). Mindfulness interventions already included in the basic program included basic rationales, mental training instructions, and psychological well-being assessment. An intensive 2-day mental training course was offered to all patients, except those with high levels of well-being.

The treatment offered to the intervention group only differed systematically from the control group in the addition of a serious gaming module (the control group did not receive something else instead); this was verified empirically. For the intervention group, the rehabilitation clinic had suitable facilitated rooms with Wi-Fi connections, tablet PCs installed with the serious game “LAKA,” and the automated planning of four 1-hour small group sessions (1-6 patients simultaneously) in connection to regular therapy hours (mostly exercise sessions at the beginning or end of a working day) during weeks 9-12. Sessions were planned for patients to have sufficient time for completing the game, at least, once. Patients logged in with their personal identification number and self-chosen password with which they also accessed Web surveys. Experienced therapists (3 psychologists and 1 physiotherapist) were scheduled to provide support during the first (introduction) and fourth (debriefing) sessions. The goal of debriefings was to discuss the experiences of game play and technology acceptance and to transfer learning results to patients’ daily lives. Other local staff members managed the accessibility of the game LAKA during sessions 2 and 3. Notably, patients could also download and play LAKA at home. Local therapists and other staff participated in developing their role in the delivery of serious gaming.

### The Serious Game LAKA

The serious game LAKA is an adventure game where patients take the role of an Avatar during a virtual trip around the world. The game is easy to control using a touch-screen tablet computer and takes on average 2.5 hours to complete (Multimedia Appendix 1 shows the screenshots).

In LAKA, patient players perform alternate tasks vicariously; they select optional responses in various encounters with other characters, monitor and evaluate satisfaction about selected responses (and their consequences), and meditate (3-minute exercises). First, players select between a male or female Avatar and assign a name. It was prompted that Avatar choices reflect those of the player. A cut-scene sets up the story; the Avatar, who wants change after experiencing a deterioration in physical and social functioning, meets a nonplaying character (NPC) named LAKA. LAKA challenges the Avatar to make “conscious” decisions during 16 “encounters” with other NPCs, for example, when standing in line, on getting invited to someone’s home, and at 4 destinations (ie, London, Turkey, Asia, and Africa) on a trip around the world. Each “encounter” is built as a flow of Avatar actions and NPC responses.

For each Avatar action, 1 of 5 options (eg, physically interact, verbally react, or ignore something) can be preselected and confirmed by players. These options are modeled after a set of reference values—generosity, moral discipline, forbearance, and enthusiastic perseverance. NPC responses are unpredictable, for example, a friendly act can result in a kind response or being scammed. At the end of each destination, LAKA asks the Avatar to self-rate the level of “satisfaction” regarding his or her choices. Indirect feedback, in the form of a number of puzzle pieces, is given by (1) the degree of correspondence of Avatar choices with the reference values and (2) the degree to which that correspondence agrees with satisfaction ratings. When the Avatar is depicted “mind-wandering” when traveling across destinations, instructions are received for a basic meditation exercise (focused attention and open monitoring) [50]. These model-based elements are interspersed with short action games, puzzle games, images, and information associated with the location of the Avatar to be enjoyed or skipped by preference.

### Quantitative Measures

#### Outcomes

Table 2 provides an overview of the outcome variables, surveys with references to instrument validity information, and times of assessment. Available primary outcome measures for operationalizing elements of research questions 1 and 2 included 4 evidently valid numerical rating- and Likert-scale measures that operationalize relevant and plausible targets for mindfulness-based intervention in the target group [11,48]: a numerical rating scale for the current pain intensity, the Checklist Individual Strength (CIS) for fatigue, the catastrophizing subscale of the pain coping and cognitions list, and the Symptoms Checklist (SCL-90) for psychological distress [51-54]. In addition, Likert-scale items on patients’ global impression of change (PGIC), general health and functioning, and treatment satisfaction were available to operationalize secondary outcomes. The PGIC was measured using a single ordinal scale item [48]. Three available 0-100 numerically scaled questionnaire items about perceived health and functioning formed an internally consistent scale (Cronbach alpha at t1=.80, at t2=.75) for general subjective health and functioning. Furthermore, 3 different questions assessed the treatment satisfaction, 2 of which were taken from the consumer quality index for rehabilitation centers [55].

#### Adherence

The operationalization of adherence distinguishes between progress in game play and debriefing attendance. The latter was established from clinical recordings of presence at either the initially planned or a rescheduled debriefing session. Log data from the game, designed to track the progress and give feedback on the performance, were used to determine the categories of completion percentage. These categories (5-1) represent 0%-6% completion when no progression logs were observed and <50%, 50%-75%, 75%-100%, and >100% game completion when patients played the game LAKA more than once.

**Table 2:** Primary and secondary outcome measures.

Variables		Survey information	Time^a^
**Primary outcomes**			
	Pain intensity	Current pain intensity Numerical Rating Scale 0-100 [53]	t0, t1, t2
	Fatigue	Checklist Individual Strength [51]	t0, t1, t2
	Psychological distress	Symptom Checklist [52]	t0, t1, t2
	Pain coping and cognition	Pain Coping & Cognitions List; catastrophizing subscale [54]	t0, t1, t2
**Secondary outcomes**			
	Global impression of change, general health, and functioning	How do you assess your health, compared with the situation at the start of your treatment? (−2, much, or −1, slightly declined; 0, neither declined nor improved; 1, slightly, or 2, much improved) What do you think of your current health in general? (0, bad-100, excellent) Please indicate how satisfied you are generally taken with your current level of functioning. (0, not at all satisfied-100, very satisfied) Please indicate the distance from your “old” level of functioning before the onset of the complaint. (0, very far removed-100, not at all removed)	t1, t2
	Treatment satisfaction	Would you recommend this treatment center to other rehabilitation patients? (1, certainly not; 2, probably not; 3, probably yes; 4, certainly yes); item from the consumer quality (CQ) index [55] Which grade would you give to the rehabilitation center? (0-10; CQ-index) Did the treatment meet your expectations? (1, not at all; 2, mostly not; 3, mostly; 4, completely)	t2

^a^t0=baseline; t1=intermediate (after 8 weeks of treatment); t2=posttreatment (after 16 weeks of treatment).

#### Case Description and Potential Confounders

Data retrieved for describing patients and enabling the optimal control for observed confounding factors consist of demographics, medical history, physical and psychological examination results, and registrations of allocated and attended interventions.

### Study Size

Study size was determined by *a priori* power calculation as described in detail in the protocol [15]. G*Power was used to calculate a required sample size of 212 patients. These calculations were based on a multivariate analysis of variance of global effects (1−beta=0.8, *f*^2^=0.0625, alpha=.05, 2 groups, and maximally 5 outcomes). By taking a margin of 20% for dropout and missing values into account, the minimum number of patients was finally determined at 250.

### Statistical Methods

All statistical analyses were performed using SPSS 22 (IBM, New York). Descriptive statistics and chi-square and Student *t* tests were used to summarize demographic, disease-specific, treatment exposure, and baseline outcome characteristics. Variables that may differ per group on the baseline were added as covariates in subsequent analyses. Statistical methods were generally aimed at testing two-sided hypotheses regarding study group differences in differences between intermediate and posttreatment outcome levels as this corresponds with the timing of the additional serious gaming intervention. Furthermore, Sidak-Holm correction was used when controlling for multiple outcome testing [56].

First, a multivariate mixed linear effect model was fitted to estimate a parameter for the study group difference in simultaneous change of the 4 primary outcomes between intermediate and post treatment assessments. For this, the MIXED procedure for defining parallel growth processes was applied on standardized scores of the 4 primary outcome variables (Textbox 1) [57]. The procedure facilitates an intention-to-treat analysis, optimizes statistical power, imposes an equality constraint on parameter estimates across multiple outcome measures, and takes outcome interdependencies into account. Hereby, the parameter estimation is unbiased under the assumption of missing data at random. To correct the parameter estimate for design limitations, it was modeled together with components that are not logically attributable to the serious gaming intervention, including global time and group effects, group differences in outcome changes in time prior to serious gaming, and covariates. Furthermore, selections between nested models, that is, excluding or including factors for time, treatment sites, and covariates were based on the statistical significance of changes in the model fit.

**Linear mixed modeling operations.**

**Operational details on the (planned or initial) multivariate linear mixed model:**

- All models applied the restricted maximum likelihood estimation.
- MIXED requires a vertical (re)structured dataset with all outcome values inserted in one column, nesting primary outcomes (4) and time factors (3) within individuals.
- Indexes were created for individuals (1-275), outcomes over time (1-12), outcomes (pain intensity=1, fatigue=2, catastrophizing=3, and psychological distress=4), time (baseline=1, post=2, and intermediate=3 [reference category]), and group (1=intervention, and 2=control [reference category]).
- The procedure used standardized outcome values, calculated separately within outcomes.
- An unstructured covariance matrix (UN) for the random effects and a heterogeneous autoregressive matrix for the repeated effects were assumed when fitting multivariate models. Use of alternative covariance structures (UN, compound symmetry, autoregressive, Toeplitz, and ante dependent) either disabled convergence or resulted in worse fit.
- Basic model specification: the outcome index was specified as randomly varying for the estimation of intercepts for each of the 4 outcomes. The planned “basic” model contained 11 fixed-effect parameters, including 4 outcome factors, 3 treatment sites, 2 time factors (1=intermediate vs baseline, 2=intermediate vs post), and 2 group × time factors (group × time 1, group × time 2), with random error terms (10) and repeated effects (13), adding up to 34 parameters to be estimated in total.
- Model fit changes, that is, exclusions (ie, site and time factors) or inclusions (covariates), were assessed using (chi-square) tests for differences in the −2 Log Likelihood information criterion.
- Sensitivity analyses revealing similar results included multivariate models run on full cases only, outcome data after outlier removal (*z*-scores above 3 or 5), and alternative *z*-score calculations.

**Operational details for univariate mixed linear models:**

- An unstructured covariance matrix was assumed for all univariate models.
- Univariate models included the same covariates as the multivariate models.

Subsequently, reliable change indexes (RCIs) were calculated (again based solely on the difference between intermediate and posttreatment scores) to determine within-group proportions of individual patients who reported improvement (RCI<−1.96) or decline (RCI>1.96) [58]. Improvement was defined as a clinically significant decrease (RCI<−1.96) in one or more of the 4 primary outcome variables. Decline was defined as a reliable increase in one or more outcomes (RCI>1.96). When patients did not show decline or improvement, their status was deemed stable. Differences in proportions in these categories were compared between the groups.

Second, effects of serious gaming on particular plausible outcome types identified through qualitative research were estimated using univariate mixed linear effect models of unstandardized outcomes. Third, (changes in) secondary outcomes were compared between the groups. Fourth, the multivariate linear mixed model was rerun after replacing the original group dummy variable by ordinal adherence variables to calculate parameter estimates separately for subgroups of differing rates of serious gaming progress and debriefing the attendance relative to controls (the reference category).

### Concurrent Qualitative Methods

Qualitative data consisted of patients’ typed responses to an open feedback question and audiorecorded, verbatim transcribed semistructured interviews. The open feedback question was: “How do you think serious gaming will contribute to your daily life (ranging from 0=negatively or nothing to 10=hugely)? And in what way?” We purposively selected 8 patients with varying expected contributions of serious gaming for semistructured face-to-face interviews (lasting 30-60 minutes). Of them, 2 were selected for their high expectations (scoring ≥9), 2 for their low expectations (scoring ≤1), and 4 for their mediocre expectations (4-6) regarding the contribution of serious gaming to their daily living. In addition, telephone interviews were planned with control group subjects who had been matched by direct care providers on case descriptions by gender, age, symptom patterns, and coping style. However, this was stopped after 2 short interviews (lasting <15 minutes) as it was not regarded informative due to case differences beyond the small set of matching variables. All interviews started with a request to patients to talk openly about their health status before rehabilitation and any changes experienced throughout. Subsequently, patients were invited to elaborate on the perceived contribution of serious gaming.

A deductive content analysis approach was performed on the interview transcripts using Atlas.ti. The first 4 interviews were coded independently by MV and a second author (MJ or AZ). Then, because no more differences in coding were observed, MV coded the remaining interviews. First, data were reduced by distinguishing text fragments related to patients’ expected health outcomes. To those fragments, labels were attached according to sensitizing concepts about (subjective) health outcomes because existing theoretical frameworks on relevant health outcome dimensions were available and preselected for quantitative operationalizations [51-54]. Those sensitizing concepts covered relevant outcome domains for patients with CP (physical symptoms, physical functioning, and emotional functioning (eg, anxiety and depressed mood) [54] or fatigue (subjective fatigue and motivation and concentration problems) [51].

## Results

### Participants

**Figure 1:**
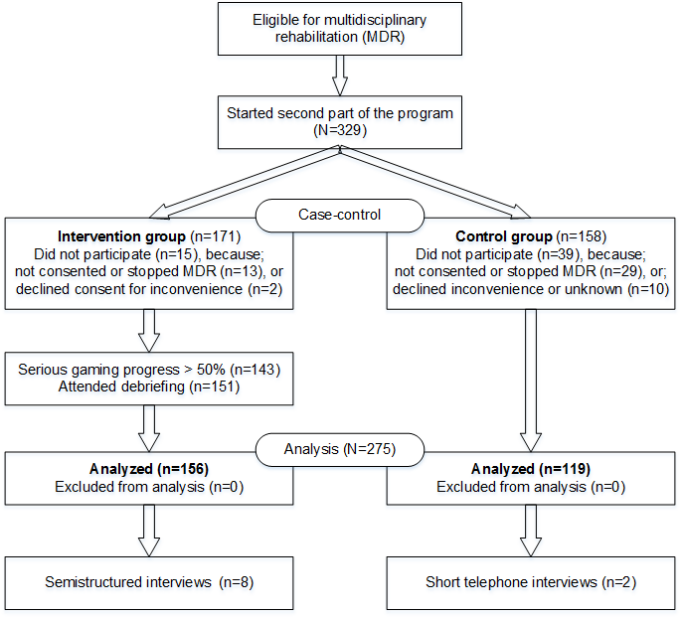
The CONSORT flowchart of participants.

Recruitment was stopped when sufficient numbers of participants were included in both study groups. By then, 83.6% (275/329) of eligible patients who had started the second part of the treatment consented to participate—156 in the intervention and 119 in the control group (Figure 1). A decline in participation due to reasons such as inconvenience (ie, presumed burdens of the treatment or study at the time of consent), delayed consent, and thereby missing patients who stopped participating during the second part of the treatment was observed more often at control sites. Furthermore, posttreatment data were missing for 1 control group patient and 7 intervention group patients.

### Descriptive Data

Back pain was the most prevalent physical symptom reported (184/275, 66.9%), but pain symptoms with other origins (headaches, gastrointestinal, fibromyalgia, and osteoarthritis) were reported as well (Table 3). Social problems were also prominent, with 49.8% (137/275) of the patients experiencing problems with family members. The modal norm group categories for the SCL-90 anxiety and depression symptom scales were “very high” relative to “healthy individuals” and “high” relative to patients with CP, but “below average” relative to psychiatric patients [52]. Compared with all 3 norm groups, modal score categories for sleeping problems were high. The study groups were similar regarding most baseline characteristics, but several *P* values found, suggesting differences between the study groups. Marginally higher socioeconomic status (SES) and more comorbid neurological and less cardiac diseases were observed in the intervention group. In the control group, relatively more patients reported back pain, were taking medication at the baseline, and had returned to work at intermediate assessment. In general, more than half of the patients had suffered from their chronic fatigue or pain condition for over 2 years before entering the rehabilitation and most had received prior (specialized) care for this (Table 3).

**Table 3:** Participants’ baseline characteristics.

Variable		Intervention group (n=159)	Control group (n=116)	Full sample (N=275)	Group difference	
					Student *t*^a^ or χ^2^ test	*P* value
Age (years), mean (SD)		44.2 (11.55)	44.9 (11.42)	44.5 (11.48)	*t*_273_=−0.5	.60
Female gender, n (%)		104 (66.7)	88 (73.9)	192 (69.8)	χ^2^_1_=1.7	.19
Socioeconomic status score^b^, mean (SD)		−.05 (0.95)	−.36 (1.28)	−.18 (1.12)	*t*_272_=2.223	.03
Returned to work (at intermediate)		16 (10.3)	24 (20.2)	40 (14.5)	χ^2^_1_=5.4	.02
**Highest educational level (N=132)^c^, n (%)**					χ^2^_3_=2.1	.56
	Primary school	1 (1.3)	0 (0.0)	1 (0.8)	
	Lower secondary education	21 (27.3)	12 (21.8)	33 (25.0)	
	Higher secondary education	28 (36.4)	19 (34.5)	47 (35.6)	
	Tertiary education	28 (36.4)	23 (41.8)	51 (38.6)	
**Work status, n (%)**					χ^2^_3_=1.6	.67
	Full-time employed	24 (15.1)	18 (15.5)	42 (15.3)	
	Fully absent	55 (34.6)	48 (41.4)	103 (7.5)	
	Partially absent	39 (24.5)	31 (26.7)	70 (25.5)	
	Unemployed	38 (24.4)	22 (18.5)	60 (21.8)	
**Pain locations, n (%)**					
	Neck or head	77 (49.4)	61 (51.3)	138 (50.2)	χ^2^_1_=0.1	.76
	(Low) back	92 (59.0)	92 (77.3)	184 (66.9)	χ^2^_1_=10.3	.001
	Upper extremities	57 (36.5)	44 (37.0)	101 (36.7)	χ^2^_1_=0.0	.94
	Lower extremities	59 (37.8)	57 (47.9)	116 (42.2)	χ^2^_1_=2.8	.09
**Symptom duration, n (%)**					χ^2^_3_=1.9	.60
	3-6 months	13 (8.4)	10 (8.4)	23 (8.4)	
	6-12 months	35 (22.6)	20 (16.8)	55 (20.1)	
	1-2 years	36 (23.2)	26 (21.8)	62 (22.6)	
	>2 years	71 (45.8)	63 (52.9)	134 (48.9)	
**Symptom course, n (%)**					χ^2^_2_=0.1	.95
	Deteriorating	100 (4.1)	76 (63.9)	176 (4.0)	
	Improving	23 (14.7)	19 (16.0)	42 (15.3)	
**Presence of comorbid medical diagnoses, n (%)**					
	Cardiology	19 (12.2)	25 (21.0)	44 (16.0)	χ^2^_1_=4.0	.048
	Neurology	16 (10.3)	0 (0.0)	16 (5.8)	χ^2^_1_=13.0	<.001
	Endocrinology	14 (9.0)	10 (8.4)	24 (8.7)	χ^2^_1_=0.0	.87
	Pulmonology	24 (15.4)	19 (16.0)	43 (15.6)	χ^2^_1_=0.0	.90
**Visits to other health care providers during the program, n (%)**					χ^2^_4_=1.7	.79
	Never	54 (36.2)	44 (37.3)	98 (36.7)	
	1-2 times	49 (32.9)	37 (31.4)	86 (32.2)	
	3 times or more	46 (30.9)	37 (31.3)	83 (31.1)	
Body mass index (kg/m^2^), mean (SD)		27.0, 5.16	27.1, 5.13	27.1, 5.13	*t*_270_=−0.181	.86
(Very) low oxygen absorption capacity (Åstrand class)^d^, n (%)		44 (38.3)	36 (42.3)	80 (40.1)	χ^2^_1_=3.0	.81
Symptom recurrence (yes), n (%)		93 (59.6)	73 (61.3)	166 (60.4)	χ^2^_1_=0.1	.77
Previous specialized medical care received (yes), n (%)		101 (64.7)	85 (71.4)	186 (67.6)	χ^2^_1_=1.4	.24
Treated elsewhere (baseline), n (%)		81 (52.3)	64 (53.8)	145 (52.9)	χ^2^_1_=0.1	.80
Medication intake, n (%)		104 (66.7)	93 (78.2)	197 (71.6)	χ^2^_1_=4.4	.04

^a^If Levene’s test for equality of variances was significant, equal variances were not assumed.

^b^The socioeconomic status (SES) index by neighborhood is derived from a number of characteristics of the people living there: their education, income, and position in the labor market. The higher the index, the higher the SES. Nationally, the mean is 0, SD is 1.09, and the minimal and maximal scores are −6.75 and 3.06, respectively.

^c^Highest education data were incomplete because it was not administered for a part of the course of the natural experiment; missing values (N=143) were group independent.

^d^Physical condition: age- and weight-corrected oxygen absorption capacity measured using the submaximal Åstrand performance test. Missing values are because of the exclusion of observations under 120 beats per minute or testing contraindications (ie, high blood pressure).

### Qualitative Results

Codes describing intervention group patients’ (4 males, 4 females) responses to open questions about expected outcome changed because serious gaming did not contain the domains of physical symptoms, physical functioning, or subjective fatigue. However, in interviews with patients with the highest expectations (score 8 or 9 out of 10; 2/8, 25%), possible benefits in the realms of emotional functioning (ie, depressed mood and obsessive or compulsive behavior) and concentration problems were voiced. These outcome domain labels were attached to patients’ expressions about expectations of improved awareness, regulation, or transcendence of negative thought and lack of interest (depressive mood), problems in decision making (obsessive or compulsive behavior), or losing focus on tasks (concentration problems), which is (partly) illustrated by the following quotes:

What I gain from it? Yes, maybe that when you're busy with something…that you're really focused on it and not being distracted…Yes, it's clear that I have that focus more.

In your daily life you are confronted with things that you, or I in any case, did not initially see as stress…Well, I often travel by train, and sometimes things annoy me, but I usually ignore it. Now I have something like: I can talk to them…So, you are irritated, and at the moment you notice it you are annoyed, so it's getting worse…Yes, you can just make it go away so that it does not adversely affect your mood.

Following these perceptions in a minority of patients, it was proposed that serious gaming generally facilitates a small amount of additional change regarding the primary outcomes of fatigue and emotional functioning (see trial registry). Moreover, additional change for patients in the intervention group was expected to be reflected by observing stronger decreases in scores based on depression and insufficiency subscales of SCL-90 and concentration problems subscale of CIS.

### Quantitative Outcome Assessment

At the baseline, participants reported on average moderate pain intensity, high fatigue, and high psychological distress levels compared with norm group averages (Table 4). After treatment, average outcome score levels were subsequently mild, higher than average, and “average” (relative to healthy norm groups).

The final multivariate mixed linear model included a study group dummy instead of the site index, SES scores, and intermediate return to work as (potential) confounding variables (Textbox 2). Model fit did not improve by adding pain location or comorbidity factors, medication intake, and amounts of particular kinds of psychotherapy received.

**Figure 2:**
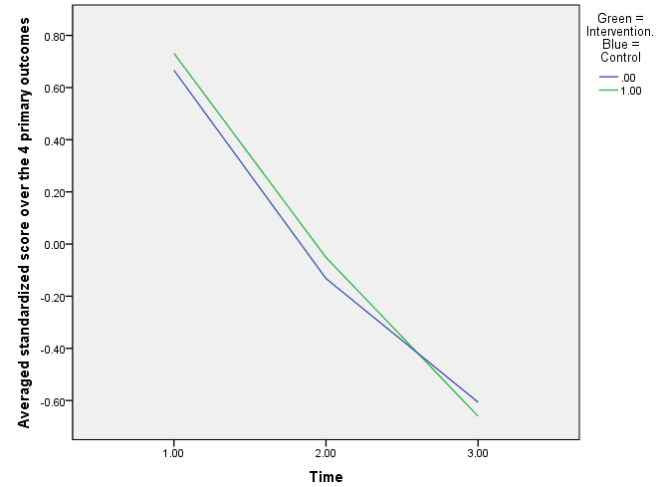
Patterns of primary outcome change during rehabilitation between the groups.

In addition, patterns of change in all 4 primary outcomes taken together throughout the rehabilitation program of each study group were visualized (Figure 2), showing that outcome scores improved in parallel before exposure to serious gaming and improved relatively more for the intervention group between intermediate and posttreatment. The multivariate mixed model, which assumed equivalent changes across the 4 primary outcomes, indicated statistically significant improvement over the first half (beta=−.805, SE=0.042, *P*<.001) and the second half (beta=−.473, SE=0.034, *P*<.001) of treatment. The parameter estimate for the interaction effect (simultaneously on the 4 outcomes) of group × time 1-2 (representing the interval between intermediate and posttreatment) favored the intervention group to a very small extent by −.119 (SE=0.046, *P*=.009); this equals to 8.59% of the total amount of outcome change within the intervention group.

**Table 4:** Primary outcome scores.

Outcome; measure and time^a^		Intervention group		Control group	
		Number of observations	Mean (SD)	Number of observations	Mean (SD)
**Pain intensity (current); Numerical rating scale (0-100) [53]**					
	t0	156	56.60 (32.27)	119	58.71 (30.92)
	t1	156	35.79 (25.80)	119	35.03 (26.31)
	t2	150	26.08 (24.07)	118	29.81 (25.56)
**Fatigue Checklist Individual Strength (CIS) [51]^b^**					
	t0	154	110.97 (18.63)	118	108.05 (15.88)
	t1	154	84.05 (26.51)	118	83.42 (24.57)
	t2	147	60.68 (27.04)	116	65.62 (26.07)
**Catastrophizing; Pain Coping & Cognitions List (PCCL) [54]^c^**					
	t0	126	3.54 (0.94)	97	3.46 (0.86)
	t1	126	2.68 (0.86)	97	2.60 (0.93)
	t2	121	2.10 (0.90)	95	2.05 (0.88)
**Psychological distress; Symptom Check List (SCL-90) [52]^d^**					
	t0	156	195.25 (50.55)	119	193.21 (49.40)
	t1	156	161.93 (43.21)	119	151.18 (35.92)
	t2	149	120.45 (32.96)	117	118.26 (28.90)

^a^t0=measurement; t1=intermediate (after 8 weeks of treatment); t2=posttreatment (after 16 weeks of treatment).

^b^Norm information for the CIS: average for healthy controls, mean=41.5, SD, 19.8; average of a norm group of patients with chronic fatigue syndrome, mean=113.3, SD, 14.6.

^c^Some data are missing by the design of routine outcome monitoring; PCCL scores are absent for very low pain intensity scores.

^d^The baseline mean is high compared with a norm group of patients with chronic pain.

**Specified multivariate linear mixed model of standardized primary outcome scores.**

A predicted (standardized) value for an individual patient on any of the 4 primary outcomes at a certain point in time is calculated as the sum of a random intercept regarding the outcome type (1=pain intensity, 2=fatigue, 3=catastrophizing, 4=psychological distress) and fixed-effect parameter estimates for the following:

- intervention group membership (1=intervention, 2=control [reference category]);
- time 0-1 (baseline=1 relative to intermediate=3 [reference category]);
- time 1-2 (post=2, relative to intermediate=3);
- socioeconomic status (SES) multiplied by the SES score;
- being returned to work at intermediate assessment;
- interaction between intervention and time 0-1; and
- interaction between intervention and time 1-2.

**Figure 3:**
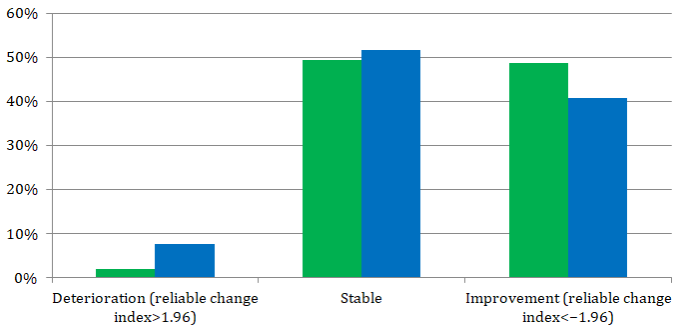
Within-group proportions for reliable improvement or decline in one or more of the 4 primary outcomes between the groups.

From the intermediate to posttreatment assessment, 48.7% (73/150) and 2.7% (4/150) of the patients in the intervention group reported reliable improvement and decline, respectively, in one or more primary outcomes (Figure 3). In the control group, these proportions were 40.7% (48/118) and 7.6% (9/118). Furthermore, proportional distributions of reliable improvement, stability, and deterioration were not different between the groups (*χ*^2^_1_=5.677, *P*=.06).

Second, univariate tests for a hypothesized group effect on changes in unstandardized CIS concentration problems and SCL depression and insufficiency scores (Table 5) resulted in a two-sided *P* value below the adjusted Holm-Sidak criterion level (alpha<.017) only for a comparatively stronger decrease in intermediate to post-SCL depressive symptom scores for the intervention group (unstandardized regression coefficient *b*=−2.74, *P*=.011).

Observations on secondary outcomes showed generally high scores for PGIC, general health, functioning (distance perceived relative to before the onset of pain or fatigue complaints and current satisfaction), and treatment satisfaction ratings (Table 6). Moreover, no group differences in secondary outcome variables were observed at posttreatment or in change since the intermediate assessment. A summary of developments in primary and secondary outcomes throughout the second part of the rehabilitation program is presented in Multimedia Appendix 2.

Finally, log data within the intervention group showed that 1 patient logged in but did not play the game, 12 played up to 50% of the game, 24 played 50%-75% of the game, 110 played 75%-100% of the game, and 9 patients continued to play a second time. Of all, 54.7% (87/156) of the patients completed 16 “encounters,” which equals to completing the game precisely once. Notably, completed encounters averaged 14.5 and ranged from 0 to 28. Among patients who did not finish the game (60/156, 38.5%), relatively few had reported completing tertiary education (5/21, 23.5%; *χ*^2^_3_=10.075, *P*=.02) or previously receiving specialist care (33/60, 55.0%; *χ*^2^_1_=4.23, *P*=.04). A debriefing session was attended by 151 patients. Groups with low adherence were too small to provide valid efficacy estimates within each.

**Table 5:** Univariate linear mixed modeling results.

Subscale used and time^a^		Intervention group		Control group		Effect	Unstandardized regression coefficient *b* (SE)	*P* value^b^
		Number of observations	Mean (SD)	Number of observations	Mean (SD)			
**Symptoms checklist depression subscale**								
	t0	156	40.49 (12.22)	119	39.87 (12.69)	t0-1^c^	−8.50 (0.92)	<.001
						t1-2	−7.20 (0.71)	<.001
	t1	156	31.99 (11.31)	119	28.89 (8.94)	t0-1 × X^d^	2.52 (1.40)	.07
	t2	149	24.85 (8.64)	117	24.50 (7.95)	t1-2 × X	−2.75 (1.07)	.01
**Symptoms Checklist insufficiency subscale**								
	t0	156	24.63 (6.68)	119	23.96 (7.15)	t0-1	−4.5 (0.48)	<.001
						t1-2	−4.0 (0.43)	<.001
	t1	156	20.1 (6.05)	119	18.62 (5.55)	t0-1 × X	.82 (0.76)	.27
	t2	149	16.11 (5.57)	117	15.97 (5.41)	t1-2 × X	−1.36 (0.65)	.04
**Checklist Individual Strength concentration problems subscale**								
	t0	154	26.69 (7.62)	118	24.71 (7.34)	t0-1	−4.84 (0.70)	<.001
						t1-2	−5.96 (0.55)	<.001
	t1	154	21.85 (7.80)	118	21.03 (7.20)	t0-1 × X	−1.25 (1.06)	.24
	t2	147	15.84 (7.88)	116	16.27, (7.24)	t1-2 × X	−1.17 (0.83)	.16

^a^t0=measurement; t1=intermediate (after 8 weeks of treatment); t2=posttreatment (after 16 weeks of treatment).

^b^Sidak-Holm-corrected alpha criterion levels were applied to the 3 primary outcomes, being .02 for the lowest *P* value, .03 for the second highest *P* value, and .05 for the highest *P* value.

^c^t1 parameters in this table are multiplied by −1 because t1 (index=3) was the reference category.

^e^X: intervention group.

**Table 6:** Secondary outcomes by group and time (t1=intermediate [after 8 weeks of treatment]; t2=posttreatment [after 16 weeks of treatment]).

Outcome		Intervention group			Control group				∆ Group (by time)^a^	
		t1 (n=156)	t2 (n=150)	t1 (n=119)		t2 (n=118)	Student *t* or χ^2^ test			*P* value
**Patient global impression of change, n (%)**								*χ*^2^_6_=3.3		.77
	Much deteriorated	1 (0.1)	0 (0.0)	1 (0.1)		1 (0.1)			
	Slightly deteriorated	5 (3.2)	4 (2.8)	2 (1.7)		5 (4.3)			
	Stable	16 (10.3)	10 (6.9)	25 (21.0)		6 (5.2)			
	Slightly improved	97 (62.2)	46 (31.7)	63 (52.9)		41 (35.3)			
	Much improved	37 (13.5)	85 (58.6)	28 (23.5)		63 (54.3)			
**Subjective health and functioning, mean (SD)**								*t*_266_=−1.16		.25^b^
	General health	55.45 (24.18)	71.23 (22.57)	57.97 (23.17)		71.90 (20.39)			
	Functioning “level”	46.05 (25.81)	70.19 (25.22)	50.02 (25.66)		69.78 (24.53)			
	Functioning “distance”	40.12 (25.34)	55.47 (32.10)	42.04 (27.65)		52.85 (31.37)			
**Treatment satisfaction, mean (SD)**									
	Rating program	N/A^c^	8.33 (1.20)	N/A		8.06 (1.46)	*t*_266_=1.65			.10
**Recommend program to other patients, n (%)**								*χ*^2^_2_=4.8		.09
	Certainly not	N/A	0 (0.0)	N/A		0 (0.0)			
	Probably not	N/A	4 (2.7)	N/A		9 (7.6)			
	Probably yes	N/A	40 (26.7)	N/A		37 (31.4)			
	Certainly yes	N/A	106 (70.7)	N/A		72 (61.0)			
**Expectations met, n (%)**								*χ*^2^_3_=4.67		.20
	Not at all	N/A	0 (0.0)	N/A		3 (2.5)			
	Mostly not	N/A	14 (9.3)	N/A		12 (10.2)			
	Mostly	N/A	84 (56.0)	N/A		58 (49.2)			
	Completely	N/A	52 (34.7)	N/A		45 (16.8)			

^a^If data were available at intermediate and posttreatment, group differences were assessed in change from intermediate to posttreatment.

^b^Group differences were tested in a change of the average scores over the sums of three items (that together formed an internally consistent scale); similar results were obtained if MIXED or repeated measures analysis of variance was used.

^c^N/A: not applicable.

## Discussion

### Summary of Evidence

In this study, we aimed to determine to what extent and in what respect a novel 4-hour mindfulness-based serious gaming intervention is effective in facilitating additional change in relevant physical and emotional functioning outcomes during a regular multidisciplinary rehabilitation for patients with CP or FSS. Furthermore, we studied whether such effects have clinical relevance for health improvement as conceived by patients themselves and whether these effects depend on the varying adherence within a regular care setting. Patients with mainly (low) back pain with comorbid psychosocial problems were found to adhere well to additional serious gaming during regular multidisciplinary rehabilitation, resulting in a very small (merely statistical) strengthening effect on the reduction of physical and emotional symptoms, as a whole, and of depressive symptoms, in particular. The effect of serious gaming alone, as a relatively small additional program component, did not reach clinically relevant levels; this was also suggested because patient impressions of health change and treatment satisfaction showed no improvement compared with the regular program, which already showed high satisfaction and treatment success rates. Nonetheless, within this context of multidisciplinary rehabilitation, 4 additional hours planned for serious gaming (4% of therapy time) in small groups, largely without direct professional support, accounted for 8.9% of the total average primary outcome change for the intervention group during rehabilitation.

Several insights arise from relating these results to those of previous studies on similar interventions. First, the very small effect size found in this study suggests a relatively weak effect compared with the small effect sizes found in previous studies. Those studies included evaluations of the effect of exposure to games on health outcomes with pragmatic trial designs [59,60], as well as systematic reviews and meta-analyses of randomized controlled studies on the efficacy of games for various clinical and behavioral outcomes [29,34]. Plausible explanations for a lower estimate in this study are the relatively low intensity and late supply of serious gaming relative to other efficacious psychotherapy (including mindfulness) interventions offered through other modalities of multidisciplinary rehabilitation. For any such short-term component in multidisciplinary rehabilitation, an effect large enough to be generally noticeable to patients would be extraordinary within the target population, and many patients may already have benefited from “traditional” means to improve. Second, both present and earlier findings suggest that changes with mindfulness approaches occur simultaneously across outcomes [61]. Still, our quantitative and qualitative results combined also add specifically to the anecdotal evidence from previous randomized trials that depressive symptoms are a plausible target for serious gaming [60,62]. Third, our findings indicate a possible relative efficiency of the independent usage and guidance in groups that constitute a “blended” form of serious gaming. To illustrate, the effect size estimate found in this study approaches the estimates found in a previous meta-analysis on the outcomes of computer (internet)-supported therapy across chronic somatic conditions (standardized mean difference ranging between 0.17 and 0.21 across outcomes) [63]. Therefore, the results of this study indicate, but do not prove, that serious gaming could serve as a complement or substitute to (parts of) other sorts of computer-based or blended treatments aimed at allocating scarce professional guidance more efficiently. Finally, previous authors doubted whether the adherence and efficacy of computer-based interventions are readily transferable to contexts, as in this study, wherein patients are recruited from a clinical setting instead of being openly recruited from general populations via the internet or other media [64]. This study sheds light onto this transferability issue by showing that a relatively high level of adherence can be achieved within a regular health care context where self-selection for the modality is limited, when a serious gaming supplement is offered “by default,” based on understandings of usage factors [65].

### Strengths and Limitations

Strengths of this study relate to the novelty of the serious gaming approach, statistical power, and the apparently favorable conditions for pragmatic research. This evaluation addresses a unique combination of setting, patient, and intervention characteristics (mindfulness approach and blended mode of supply). Achieving the predetermined required sample size for observing a modest effect with reasonable chance responds to previous reviews on the effectiveness of games for health that found promising results for mainly small, underpowered studies [34,39]. Furthermore, this study has taken account of Type I error risk through outcome multiplicity, factors of nonusage, and risk of biased patient expectations through the informed consent procedure. In the execution of the study, we encountered occasional unintended difficulties in accessing the game (ie, forgetting passwords) but did not encountered problems or threats to internal validity, besides those inevitable and known in the protocol phase. The precision of key results was supported by the results of sensitivity analyses after outlier removals, alternate outcome standardization, removal of incomplete cases due to treatment dropout, and extensions and simplifications (eg, exclusion of baseline data, inclusion or exclusion of potential confounding variables) of the prediction model. Mixing quantitative and qualitative outcome data led to unambiguous findings regarding the size, outcome domains, and the clinical relevance of serious game effects. Regarding the external validity, this application of pragmatic methods adds complementary insight into the effectiveness of serious gaming for patients in regular health care settings beyond controlled clinical trial conditions. The inclusive patient recruitment strategy reflects the reality of a regular care setting to which the results are to be generalized.

However, several study weaknesses should be considered, comparing this study with supposedly ideal circumstances for a randomized controlled (multicenter) trial. Not applied for practical reasons were broader recruitment of treatment settings, researcher control on selection procedures, the use of an individual or site-level randomization procedure for balancing unobserved characteristics between study groups, inclusion of certain measures (long-term follow-up, objective outcomes, functional interference, quality of life, and participation), and collections of cost data. In addition, intervention group participants were aware that they received a novel treatment component. However, this is not expected to have influenced the results as an insignificant association was observed between outcome expectations of serious gaming and health outcome change levels (intermediate to posttreatment) within the intervention group. Adding an additional component to an already intensive treatment program has neither been deemed likely nor intended to increase cost-effectiveness at present, but may offer useful insight for achieving this in the future. Although previous studies have suggested that effects of serious games for behavioral change are retained [29], it remains uncertain how a very small reinforcing influence on patterns of outcome change that started earlier during treatment will develop further in time. Furthermore, a lack of more stringent diagnostic methods at inclusion poses an internal validity threat. Data are also missing about characteristics of patients who dropped out during the first part of the program. Moreover, present results suggest that (everything else being equal) additional serious gaming adds very little to the outcome improvement, but intervention group participants did not reach more favorable outcome levels at posttreatment. A possible explanation is that control group symptom levels were slightly lower overall because of an effect of recruitment that was too small to observe. Besides, not all expected outcome domains found through the qualitative research were confirmed with quantitative results; this might also be attributed to a lack of power as precalculations have not been based on an increasing number of statistical tests. Finally, generalizability is limited by the convenient selection of 4 locations from a single Dutch care center.

### Suggestions for Research and Practice

In light of previous research, the very small positive effect on relevant outcomes found in this powerful pragmatic study reaffirms that both caution and optimism about the effectiveness of serious games as a treatment facilitator are warranted. Findings imply that serious gaming holds potential, as for the present mindfulness-based approach to it, but requires further investigation before wider dissemination within multidisciplinary rehabilitation programs or other regular health care settings (eg, psychological therapy). From patients’ point, expectations on potential benefits are to be placed in perspective, that is, results merely suggest that multidisciplinary rehabilitation based on a biopsychosocial approach (ie, one that includes mindfulness approaches to learning to live with CP or FSS) with generally modest effects (ie, offer little assurance for recovery) could be somewhat improved (in a slight, merely statistical, degree) by adding serious gaming as a modality. We do not suggest that additional serious gaming causes more patients to experience clinically relevant treatment effects. Nonetheless, the study results do suggest that the delivery of a small part of an evidence-based treatment by means of a serious game can be trusted. Therefore, researchers should continue to pursue adequately powered and, if possible, RCTs when aiming to assess the effects of (mindfulness-based) serious gaming.

As part of a general search for effective combinations of approaches, techniques, and modalities to intensive rehabilitation programs, the serious gaming approach requires further theoretical refinement as to know how and when clinically relevant benefits are achieved by which patients and why. The current state of evidence provides little support as to identify those circumstances in which patients with CP or FSS will likely have best experiences and outcomes from which (computer-based) biopsychosocial or alternative treatments and why [10,18,66,67]. In this regard, our findings specifically point toward very small positive effects when (mindfulness-based) serious gaming is presented later on in a rehabilitation process to patients with chronic back pain and comorbid psychosocial problems. Patients, policy makers, and professionals must be aware of the ongoing developmental stage, wherein the accumulation of knowledge is needed before the full potential of serious gaming can be realized routinely and efficiently into complex health care systems [29,68]. Thus, to achieve the highest potential of serious gaming for health, more theoretically oriented and context-sensitive studies are needed in addition to more powerful outcome assessment trials. To facilitate progress, researchers need to focus on a broad range of research questions about when and which kinds of serious games are (cost-)effective, for whom, compared with other treatment options, and why. This endeavor requires (1) hypotheses-driven process evaluations alongside trials (using quantitative, qualitative, or mixed methods); (2) transparent and universal reporting on the qualities of the methodology (ie, eHealth CONSORT statement additions [69]), serious games for health (rationale, functionality, and data security [70]), and behavioral change content (theoretical approaches, change strategies, and presentation methods [71]) in trials, (3) implementation research investigating organizational, professional, patient, and intervention factors; (4) impact assessment as dependent on actual reach; and (5) health technology assessments.

### Conclusions

Based on a powerful natural quasi-experiment, the results of this study suggest that serious gaming, as an additional modality for mindfulness intervention of short duration during regular multidisciplinary rehabilitation, adds very little to reducing physical and psychological symptoms in patients with CP or FSS (ie, indicated with chronic back pain and concomitant psychosocial problems). In addition, the results hint, but cannot yet prove, that these very small benefits are nonetheless relevant in terms of efficiency if one considers how little (extra) time it costs from scarce expert care providers. An effect with respect to depressive mood may exist that a minority of patients conceive as relevant for their daily life. Moreover, the findings clearly support a generally good adherence to a blended form of serious gaming in a regular care setting. Taken into account the conditions of serious gaming in this study (ie, relatively low intensity compared with the complete treatment program that patients received), the results fit the expectations created by previous studies that generally found slightly higher (small) effects on behavioral and clinical outcomes (ie, studies on serious games in various populations or studies on computer-based interventions in patients with CP or FSS). Therefore, the potential of serious games for being effective in changing behavioral and clinical outcomes across targeted populations is reaffirmed and further (theory-driven) research on serious gaming aimed at predictably (cost-)effective applications for individual patients across health care settings encouraged.

## Appendix

Multimedia Appendix 1 Screenshots and trailer.

Multimedia Appendix 2 Change in primary and secondary outcomes throughout the second part of rehabilitation.

## Abbreviations

CIS: Checklist Individual Strength
CP: chronic pain
FSS: functional somatic syndromes
NPC: nonplaying character
PGIC: patient’s global impression of change
RCI: reliable change index
RCT: randomized controlled trial
SCL-90: Symptoms Checklist
SES: socioeconomic status
