# Supplementary material for: Effectiveness of Serious Gaming During the Multidisciplinary Rehabilitation of Patients With Complex Chronic Pain or Fatigue: Natural Quasi-Experiment
Source: J Med Internet Res. 2018 Aug 15;20(8):e250. doi: 10.2196/jmir.9739 (PMC6115601; doi:10.2196/jmir.9739)
Supplement: Multimedia Appendix 1 [file jmir_v20i8e250_app1.pdf]

Multimedia Appendix 1: [Screenshots and trailer]

a) Opening screen

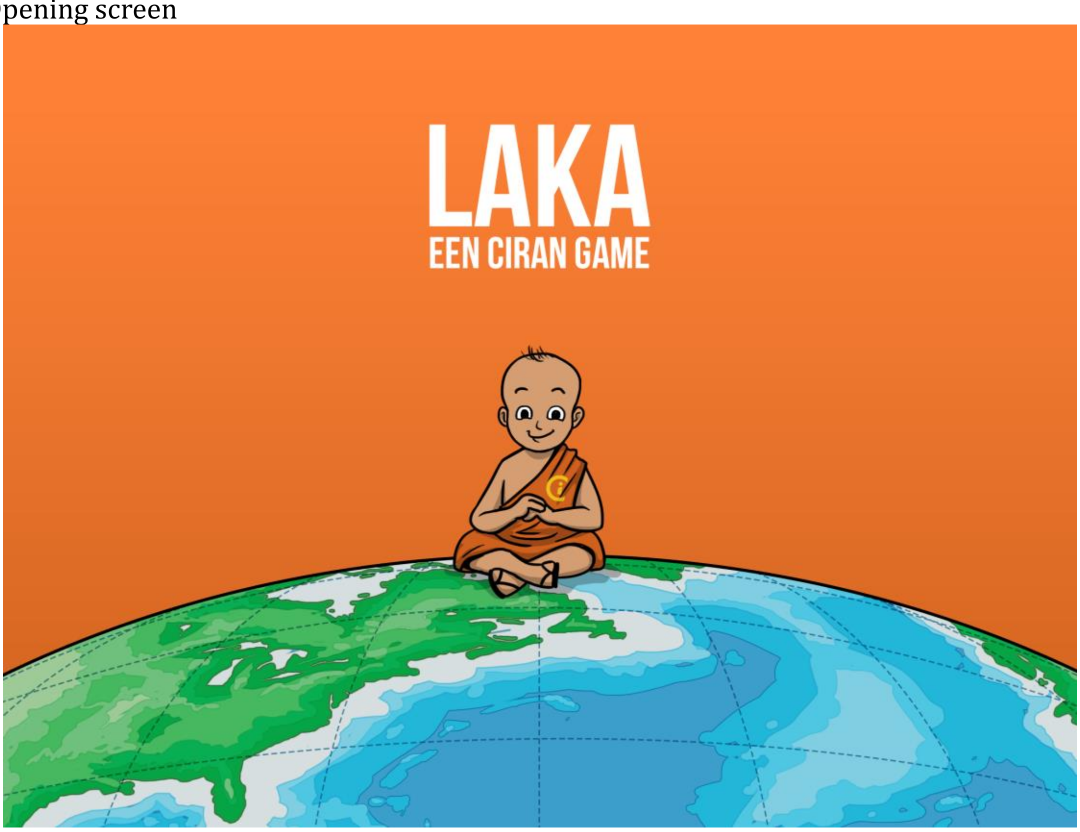

b) London Hyde Park

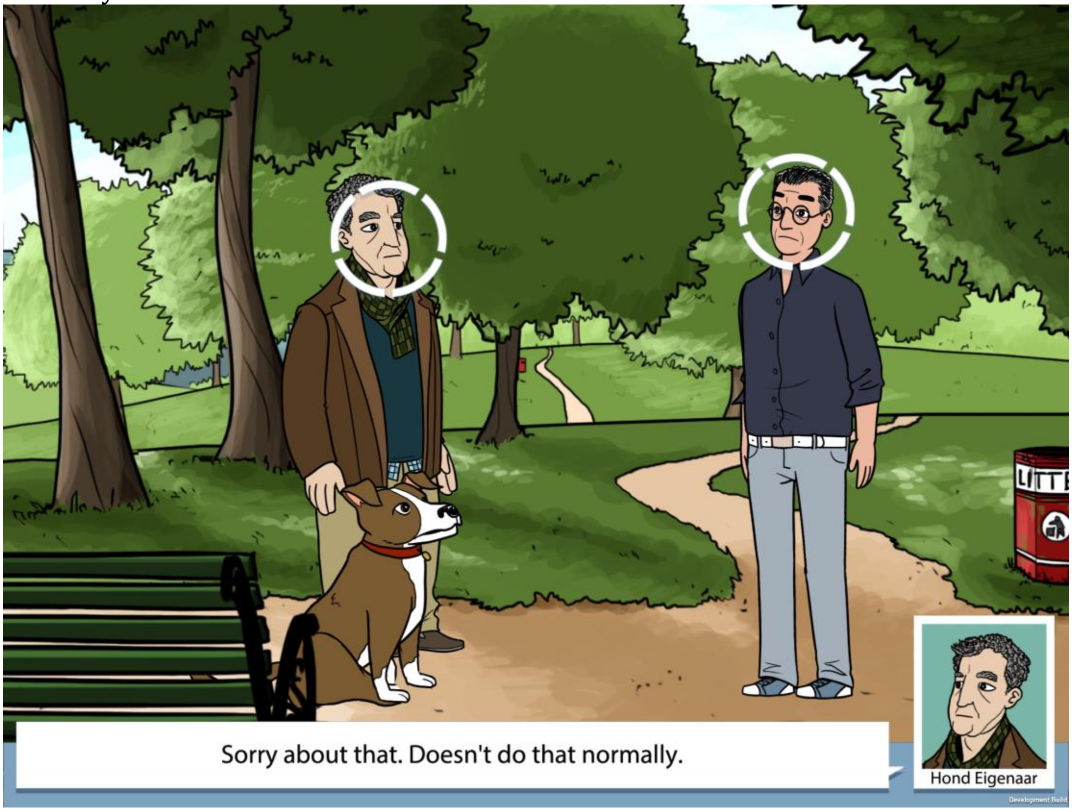

c) Asia Lhasa

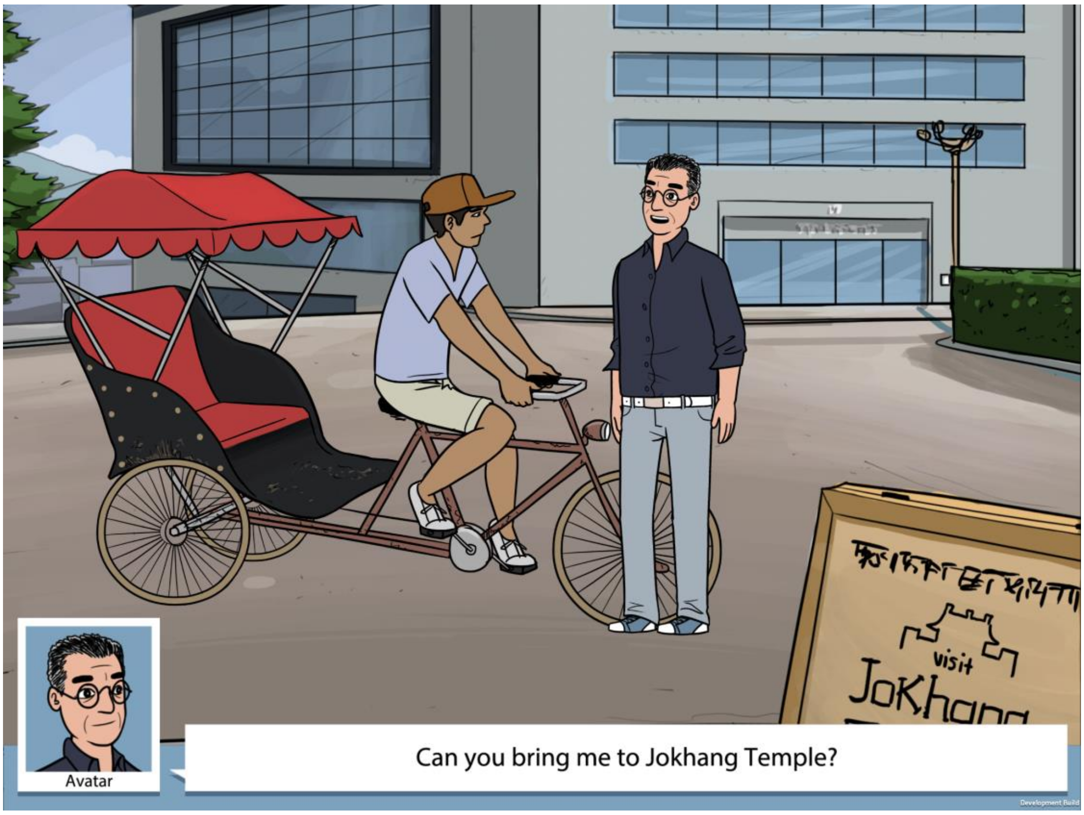

d) Puzzle screen

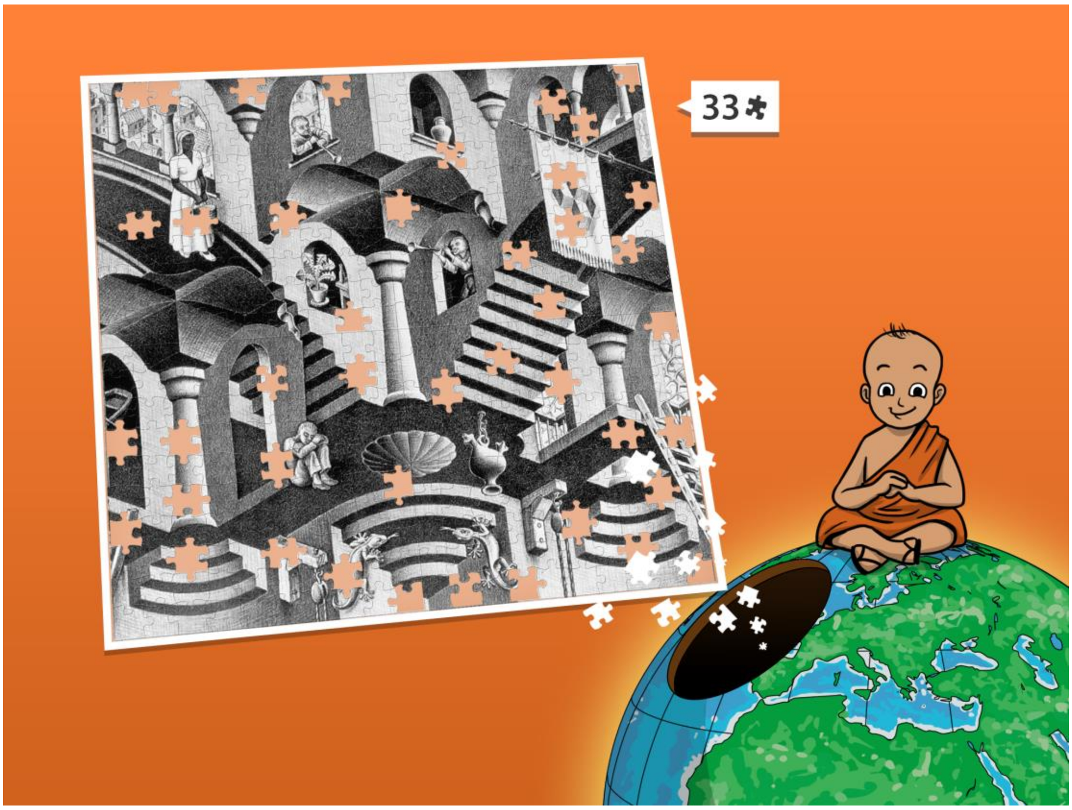

e) Protagonist prototypes

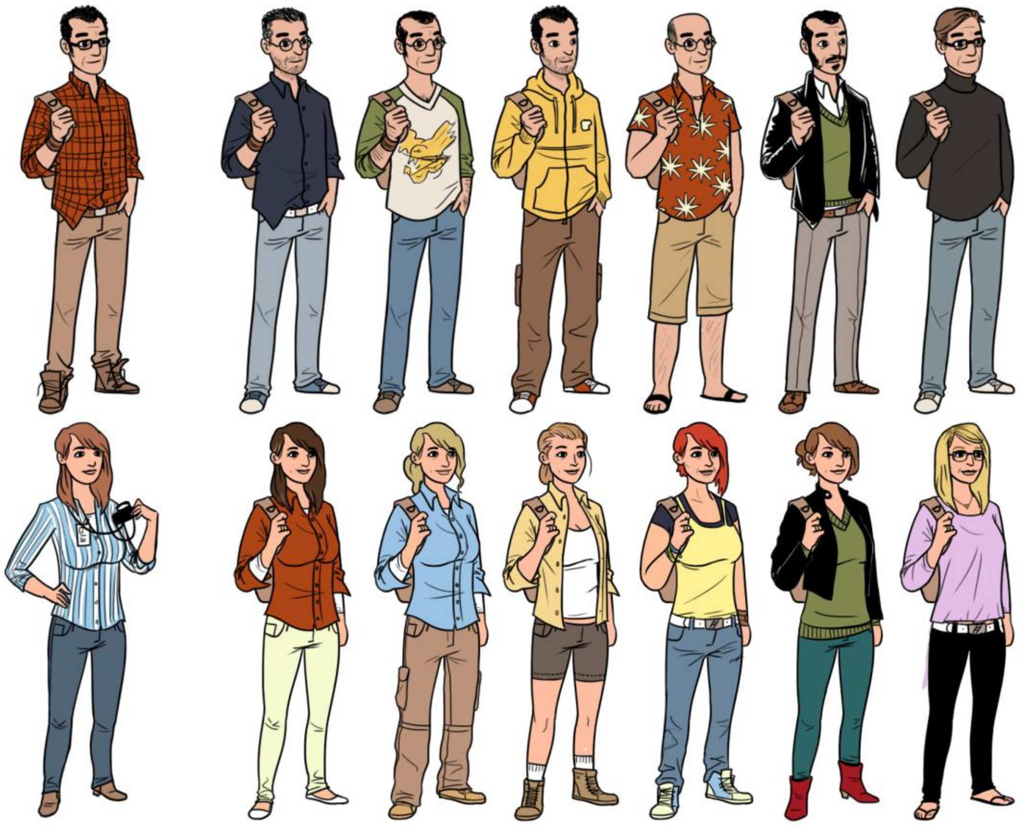

f) Laka prototypes

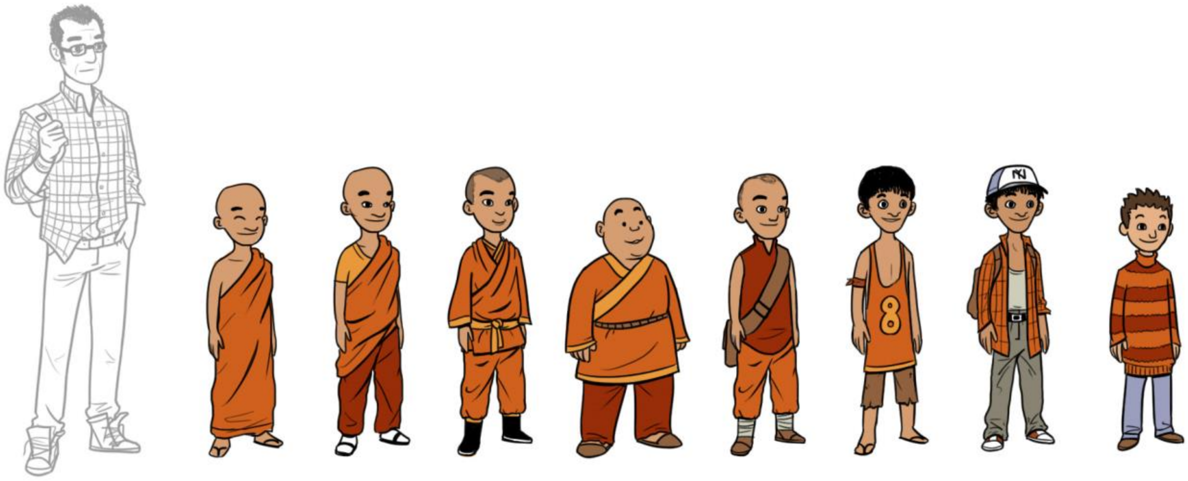

Trailer of the experimental serious gaming intervention (Dutch, English transcript below):

https://www.youtube.com/watch?v=gcRidFTk_7w

Voice-over script English.

<u>General voice/ health professional</u>
Did you know that more than 7% of the general population suffers from pain for more than 3-6 months?
Chronic pain is often accompanied by fatigue and functional interference
If these symptoms persist despite good care of a GP, physiotherapist or psychologist, one could benefit from multidisciplinary rehabilitation.

We are Ciran, an institution for specialist medical care for clients with chronic pain, fatigue and functional interference.
We help clients to better manage their symptoms, and to participate as optimally as possible: at home, in social life, or at work.
For additional support in this process, we develop a serious game. We deploy this game as a part of multidisciplinary rehabilitation.
Together with our clients and scientists, we examine the contribution of serious gaming to the learning and recovery process.

<u>Patients:</u>
"A game? Why a game? Why should I do that? '
"I'm curious, Let's see what it is'
'Funny! I can do this! '

<u>General voice/ health professional</u>
The game LAKA takes you on a trip around the world. Playfully, one meets people under various circumstances.
In these encounters, you get plenty of time for making choices. Choices of which you may not be that strongly aware in daily life.
Then you may notice discrepancies between what you do in real life ... and what your ideal choices would be.
Occasionally, a wise teacher (LAKA) asks you to monitor and evaluate such choices, and introduces exercises to strengthen your self-awareness.
As such, LAKA shows your ability to be more aware of yourself and your environment, to change and adapt, discover simple sources of joy, and have valuable contacts with others.

<u>Patients</u>
"In my daily life I avoid many things ... I don't want that, but then it happens anyway! "I could concentrate well. That was pleasant. Those attention exercises, I repeated them many times... "
"Those puzzles ... I tried them 20 times. I am so persistent. I must succeed! Very frustrating...

<u>Health professional</u>
"Do things like that happen more often?"

<u>Patients</u>
"I found it difficult to handle a tablet PC! And where do you get the puzzle pieces for? Should I be rude or friendly? That depends on the situation!

<u>Health professional</u>
So you have overcame the tablet computer, and learned something. LAKA does not tell you what to do. ... You can determine it for yourself…

Between therapy sessions, physical activity and theory, LAKA provides a space to, despite chronic symptoms, play with fresh ideas for improving your quality of life and discuss them with someone else.
