# Supplementary material for: Effectiveness of Serious Gaming During the Multidisciplinary Rehabilitation of Patients With Complex Chronic Pain or Fatigue: Natural Quasi-Experiment
Source: J Med Internet Res. 2018 Aug 15;20(8):e250. doi: 10.2196/jmir.9739 (PMC6115601; doi:10.2196/jmir.9739)
Supplement: Multimedia Appendix 2 [file jmir_v20i8e250_app2.pdf]

Multi-media appendix 1: Change in primary and secondary outcomes throughout the second part of the rehabilitation program

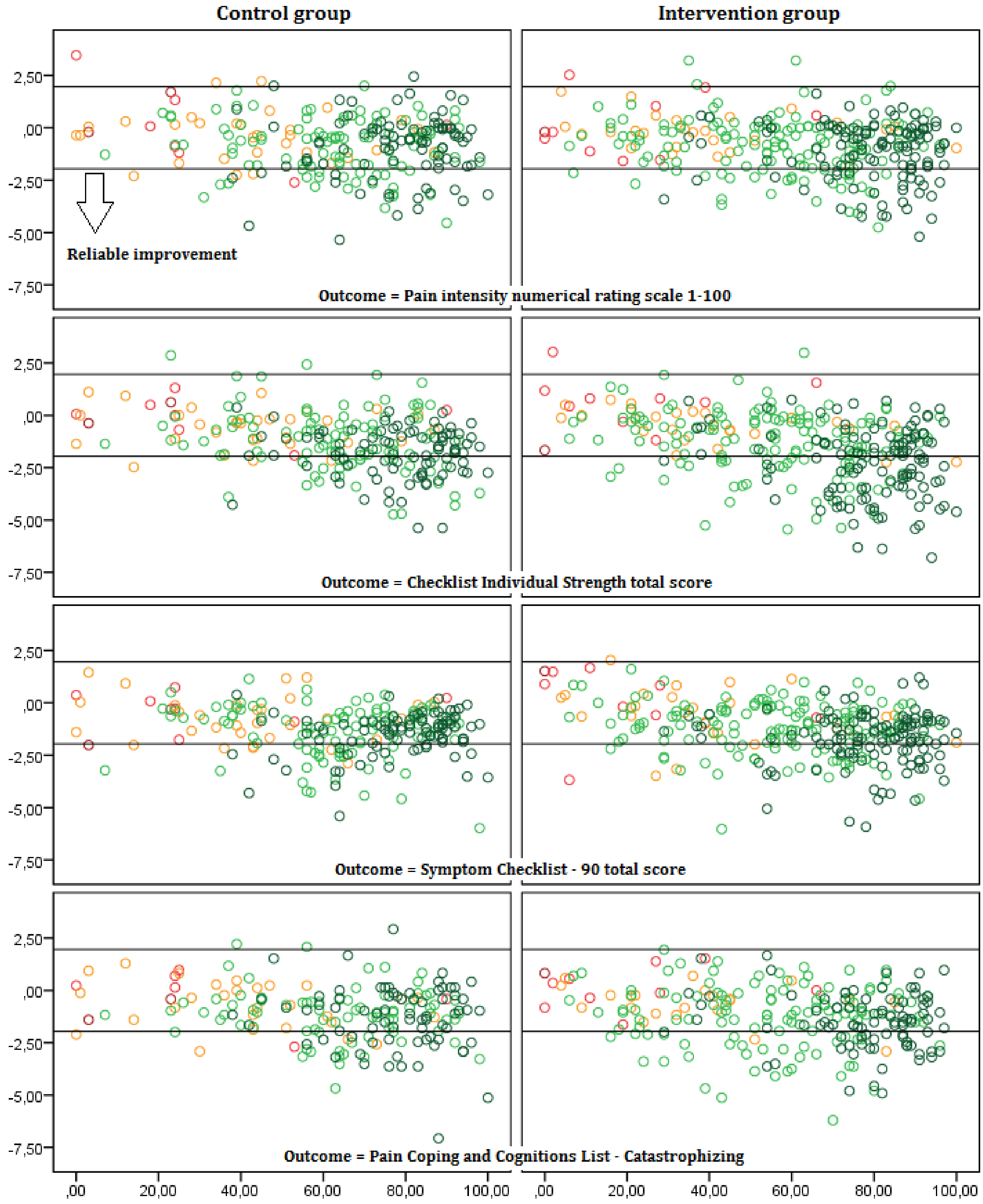

Legenda:

- Y-axis = reliable change index based on the difference between after 8 weeks of rehabilitation treatment (at intermediate) and after 16 weeks (at post)
- X-axis = General subjective health (Numerical Rating Scale 0-100)
- Color markings: Patient Global Impression of Change (PGIC) after 16 weeks of treatment; dark green = substantially improved, light green = slightly improved, orange = neither improved nor deteriorated, light red = slightly deteriorated, and dark red = substantially deteriorated.
- Left column = control group results, right column = intervention group results
- Rows represent outcome types: Current pain intensity (Numerical Rating Scale 0-100), Fatigue (Checklist Individual Strength), psychological distress (Symptoms Checklist -90), and Catastrophizing (Pain Coping and Cognitions Scale).
